# Supplementary material for: Development and Testing of the A1 Volumetric Air Sampler, an Automatic Pollen Trap Suitable for Long-Term Monitoring of eDNA Pollen Diversity
Source: Sensors (Basel). 2022 Aug 29;22(17):6512. doi: 10.3390/s22176512 (PMC9460460; doi:10.3390/s22176512)
Supplement: Supplementary file 1 [file sensors-22-06512-s001.zip › Supplementary Materials S1.pdf]

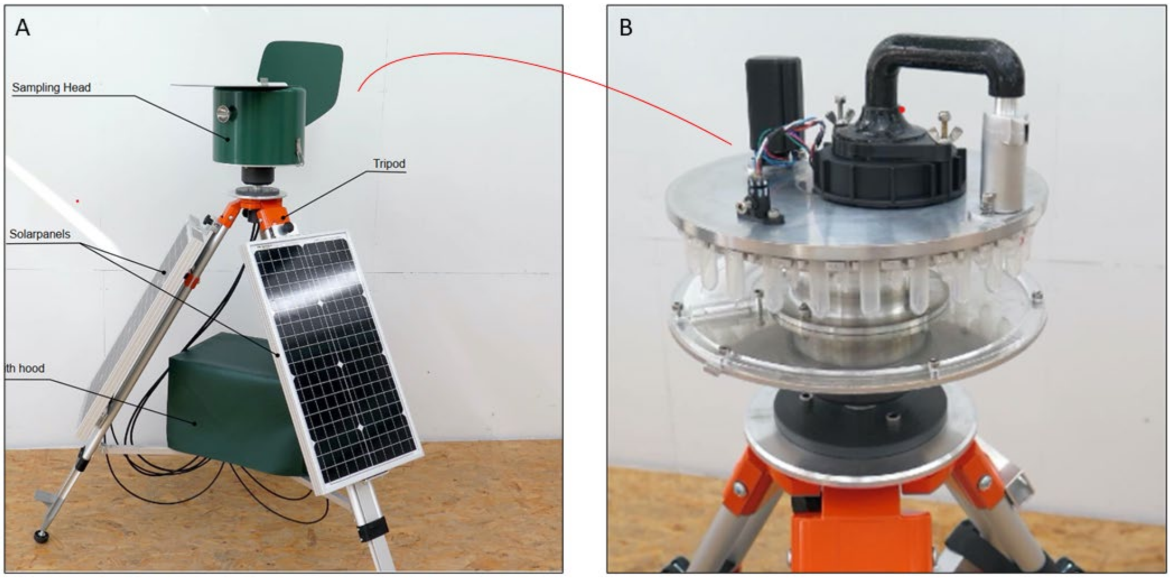


**Figure S1.** Details of the A1 volumetric air sampler: (**A**) complete details and (**B**) only the carousal**.**

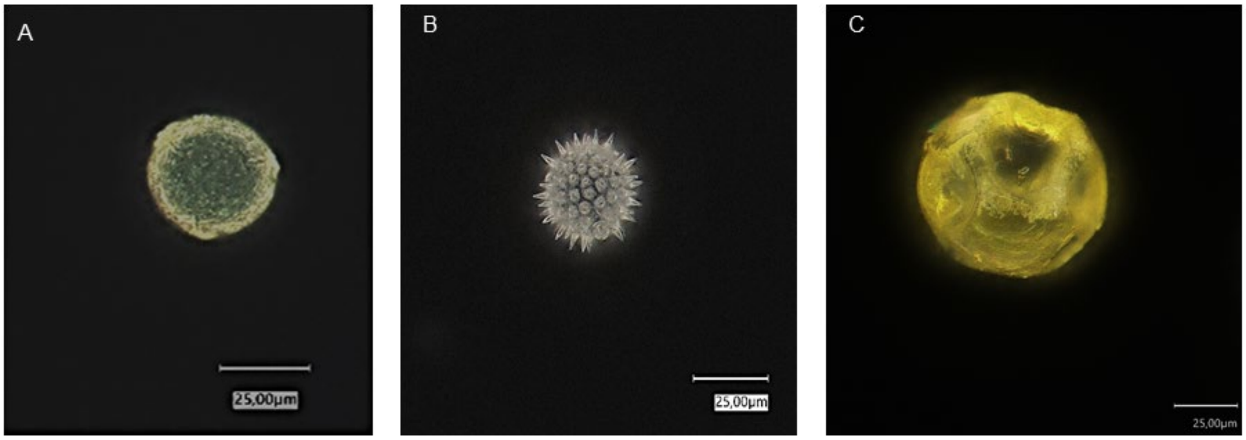


**Figure S2.** Plate showing pollen morphology and size: (**A**) *Fagus sylvatica* (European Beech), (**B**) *Helianthus annuus* (Sunflower), and (**C**) *Zea mays* (corn)**.**

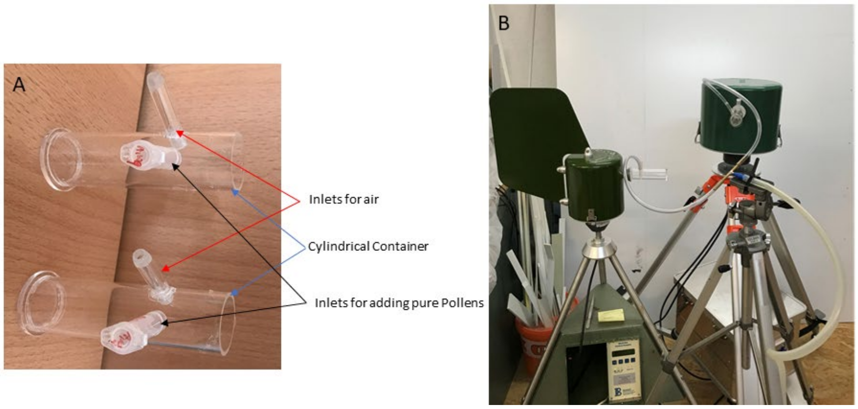


**Figure S3.** (**A**) Closed containers used in the experiment and (**B**) T. attached with the containers and the then with the air samplers**.**
